# Impact of zinc structural on the photovoltaic Properties of iron Pyrite

**DOI:** 10.1016/j.heliyon.2023.e13248

**Published:** 2023-01-31

**Authors:** Refka Sai, Ihab Shawish, Muaffaq M. Nofal, Eman A. Alghamdi

**Affiliations:** a Departement de Physique, Faculté des Sciences de Bizerte, Université de Carthage, Tunisia; b Photovoltaic Laboratory Research and Technology Center of Energy, Borj-Cedria Science and Technology Park, BP 95, 2050 Hammam-Lif, Tunisia; c Department of Mathematics and Sciences, Prince Sultan University, P. O. Box 66833, Riyadh 11586, Saudi Arabia; d Department of Physics and Astronomy, King Saud University, Riyadh 11451, Saudi Arabia

**Keywords:** Iron pyrite, Band gap, Band structure

## Abstract

$FeS_{2}$ pyrite is one of the most interesting photovoltaic materials with low-cost and natural abundance but with small band gap of 0.95 eV. In the present work, we show the feasibility of increases band gap was determined by Zinc alloying of Iron pyrite. We showed that we can increase the band gap of $FeS_{2}$ pyrite to 1.15eV by theoretical calculation and to 1.16eV using experimental method, by just adding a very small amount of Zinc (1%). We prepared our samples by chemical vapor transport technic and we utilized the technic of linear muffin-tin orbital method in the atomic-sphere approximation (LMTO-ASA). The effect of Zinc alloyed Iron pyrite were examined by transmission electron micrograph TEM, XRD, Raman spectroscopy and optical characterization.

## 1 Introduction

Iron pyrite has gained extensive research interest in the photovoltaic cell due to its high optical absorption ($∼10^{5}{cm}^{-1}\;for\;h\nu >1.4eV$) and its photocatalytic, wherever iron pyrite is beneficial for the development of high power energetic solar cells [1]. However, it is a good candidate for photoelectrochemical and photovoltaic cells due to its nontoxicity and its abundance [2,3]. It is known with photoelectric conversion lower than 3% [4]. Its band gap is almost 0.5eV less than the optimum band gap 1.4eV according to Shockley Queisser theory [5], where its experimental band gap is about $E_{g}^{\exp}=0.95\;eV$ [6] and its theory band gap $E_{g}^{th}=0.90\;eV$ [7]. Its open circuit voltage (OCV) is less than 0.2eV for that the actor for a high efficiency is limiting [8]. For that several are being because this low open circuit voltage for pyrite [[9], [10], [11], [12]], including intrinsic surface states [[13], [14], [15], [16]], the extremity of the conduction band [17] and the study of bulk defect [18].

One of the appealing methods is to reduce the cost of solar cells energy conversion to improve efficient PV cells utilizing low-cost methods of fabrication and naturally abundant materials. $FeS_{2}$ pyrite band gap is small sufficient to use in solar spectrum but is out of the way to produce a single junction PV device with highest efficiency [19].

Therefore, increasing the band gap is necessary to develop Iron pyrite-based photovoltaic. Alloying pyrite is the first feasible method to enlarge the band gap and to modulate the electronic properties of semiconductor. Altermatt et al. are the first who studied the idea of alloying pyrite [20], where they proved that doped pyrite may gain large gap to enhance the low OCV and Zn alloying is predicted as a target for future work. The band gap experimental and the band gap theory are not determined, only the band gap experimental is estimated to be 2.5eV [21]. By using density functional theory (DFT), Sun and Ceder showed that the band gap of ${FeS}_{2}$ pyrite can be enlarged by replacing some Fe by Os or Ru to have ${Fe}_{1-x}{Os}_{x}S_{2}$ and ${Fe}_{1-x}{Ru}_{x}S_{2}$. They found that the band gap Eg increase by only ∼0.1eV for x=0.5 [22]. Also, Sun and Ceder studied ${Fe}_{1-x}{Zn}_{x}S_{2}$ alloys and proved the band gap increase for x up to 0.5 [23]. Some factors are necessary to choose better cation compound for alloying such as material abundance, low-cost and environment impact that should be taking in consideration when using pyrite for PV material. Even though Sun and Ceder shown that Os and Ru alloy $FeS_{2}$ pyrite increases the band gap but note that Os and Ru are not naturally abundant [23]. The interest element that can alloy $FeS_{2}$ pyrite is Zinc because its low-cost and natural earth abundance. Due to electronic properties of Zinc chalcogenide [46,47], Zn is considered as a metal that is widely used for surface coating of semiconductor and for doping. $ZnS_{2}$ has a band gap about of 2.0 and 2.5eV [9–21], that's means the band gap of ${Fe}_{1-x}{Zn}_{x}S_{2}$ can be continuously between 0.95 and 2.5 eV. Moreover, there are some problems associated to Zn alloyed $FeS_{2}$ pyrite for band gap. First, with a small percentage of incorporated Zn, the band gap will be lower than that of Iron pyrite? Also $FeS_{2}$ pyrite and $ZnS_{2}$ share the same structure of unit cell, a second problem is to incorporate high percentage of Zn into $FeS_{2}$ pyrite, that is because of the size of ${Fe}^{2+}\;\left(0.61Å\right)$ as well as the size ${Zn}^{2+}\;\left(0.74Å\right)$.

Other transition metal doped into the iron pyrite to form alloyed iron pyrite. For example, N. Alonso –Vante et al. studied Cu-alloyed FeS2 structure phase, which is significantly affected by the deficiency of sulfur, their obtained films we can see that the conductivity p-type and the band gaps values were: 1.062–1.156 eV [51]. Nofal et al. showed that the bandgap of FeS2 increases from 0.90508 to 1.21586 eV when they replace ∼1.19% of the Fe atoms with ruthenium atoms (x = 0.0119 concentration of Ru) [52]. Sun et al. claimed that the band gap of FeS2 pyrite (0.95 eV) increases when alloys with Os to (1.2eV) [9]. Ouertani et al. [53] found that the gap of Ru-alloy can reaches (1.31 eV) where the bandgap is direct.

The main objective of this work is to understand the impact of incorporated Zn (small percentage, high percentage) in $FeS_{2}$ pyrite about its structural features while enlarging the band gap and several kinds associate to photovoltaic application. For this study, the samples were prepared by using chemical vapor transport method to enlarge the band gap around the optimal of PV performance be improve. The obtained ${Fe}_{1-x}{Zn}_{x}S_{2}$ was studied by utilizing X-ray powder, elemental analysis, and Raman spectroscopy. We studied optical and electronic properties of ${Fe}_{1-x}{Zn}_{x}S_{2}$ obtained. For our experimental work, we used the LMTO-ASA to analyze the impact of tiny concentrations of substitution Zn on the band gap of ${FeS}_{2}$ pyrite, this has been used by many researchers see Refs. [7,[40], [41], [42], [43], [44], [45]].

## 2 Experimental

### 2.1 Materials

Pyrite crystal is fabricated by using chemical vapor transport, where we used bromine as transporting agent in a silica ampoule with a diameter of 25 mm and length of 150–200 mm for temperature gradient from 850to950K [24]. The ampoule contained 3 g of ${FeS}_{2}$ pyrite powder. We added 5% ZnS powder to this element. This element can formally be explained by using a molar fraction ratio between ${FeS}_{2}$ and FeS. In three ways ZnS powder can be employed: (i) It absorbs metal impurities in growth system [25]. (ii) It constructs solid solutions with iron monosulfide [26]. (ii) It is inserted into the growing pyrite crystals [27]. ZnS and pyrite are transported by bromine, the chemical transport reactions can be described by the following Eqs. (1)–(1)–(3)(1)–(3):

(1) $$1.5\;{Br}_{2}\left(g\right)+{FeS}_{2}\left(s\right)↔\;S_{2}\left(g\right)+{FeBr}_{3}\left(g\right)$$

(2) $$2{FeBr}_{3}\left(g\right)+{FeS}_{2}\left(s\right)\;⟷\;S_{2}\left(g\right)+3{FeBr}_{2}\left(g\right)$$

(3) $${Br}_{2}+2ZnS\;\left(s\right)\;↔\;S_{2}\left(g\right)+2ZnBr\;\left(g\right)$$

The transport time is between 175h and 200h. From the above equations we deduce that the presence of ZnS in the growth system makes Zn− and S− more stable in the pyrite crystals. During the transport in the presence of a ZnS, we obtained Zn-dopped ${FeS}_{2}$ pyrite crystals. In the process of the growth, we used a big quantity x(x=0,1,2,6.1,15and50%) of ZnS. After all reactions with iron sulfide, we obtained p-type ${Zn}_{x}{Fe}_{1-x}S_{2}$.

### 2.2 Characterization

**Fig. 1:**
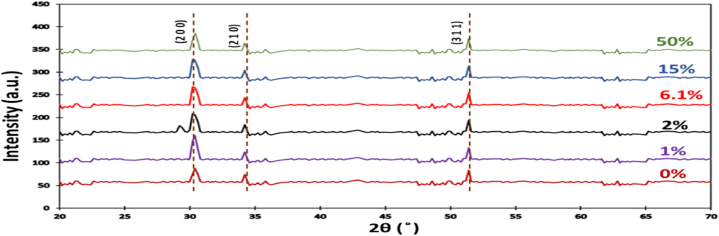
XRD patterns.

For the structural investigation, (XRD) X-ray diffraction was determined by using Philips diffractometer with CuK α radiation for λ=1.5412Å . Raman spectra are recorded using an organ ion laser Jarrel-ASH 00-25-300 Raman spectrophotometer to further analysis of the phase evaluation with increasing Zn. Transmission Electron Microscope (TEM) was utilized to determine the morphology of *p-type* ${Zn}_{x}{Fe}_{1-x}S_{2}$ using a JEOL 2010 microscope. The optical absorption spectra of p-type ${Zn}_{x}{Fe}_{1-x}S_{2}$ pyrite obtained by using a SHIMADZU 3100s spectrophotometer. Fig. 1 shows the XRD peaks at 2θ angles to the crystal planes (200), (210) and (311) are basically analyze that pyrite structure (JCPDS card n ∘ 05-1375) which no noticeable impurity peak was observed. We have three strongest peaks at (200), (210) and (311). The three typical diffraction peaks may be present description of the decreases of the crystallin size with an increasing Zn amount. This confirm that the incorporation of Zn into $FeS_{2}$ pyrite should increase of the lattice constant value. The cell parameters were carried out the Rietveld method by using PDXL program, are listed in Table 1 below. Powder X-ray diffraction measurements of ${Zn}_{x}{Fe}_{1-x}S_{2}$ showed that our crystals ${Zn}_{x}{Fe}_{1-x}S_{2}$ exhibit cubic structure in pyrite phase. Hence ${Zn}_{x}{Fe}_{1-x}S_{2}$ has structure pyrite belonging to the space group $Pa\bar{3}\;\left(205\right)\text{.}$ Its parameters (lattice constant a, structure ν which control the position of Sulfur atoms, gap energy experimental and composition) are in Table 1. Our XRD show that the alloy of Zinc in Iron pyrite leads to the increases of the lattice constant. Also, we mark that Zn alloy $FeS_{2}$ pyrite of 50 % did not have any impact on the level of alloy, that indicate is not the limiting level of alloying. Hall measurements and conductivity revealed that all crystals were p-type.

**Table 1:** Exprimental output.

ConcentrationofZn%	0	1	2	6.1	15	50
Latticeparametera(Å)	5.415	5.416	5.417	5.419	5.504	5.616
Structureν	0.1001	0.101	0.1015	0.1018	0.1021	0.1053
Temperature(°C)	780	800	850	870	900	950
composition	$FeS_{2}$	${Zn}_{0.01}{Fe}_{0.99}S_{2}$	${Zn}_{0.02}{Fe}_{0.98}S_{2}$	${Zn}_{0.061}{Fe}_{0.939}S_{2}$	${Zn}_{0.15}{Fe}_{0.85}S_{2}$	${Zn}_{0.5}{Fe}_{0.5}S_{2}$
BandgapexperimentalEg(eV)	0.95	1.16	0.73	0.72	0.71	0.70

**Fig. 2:**
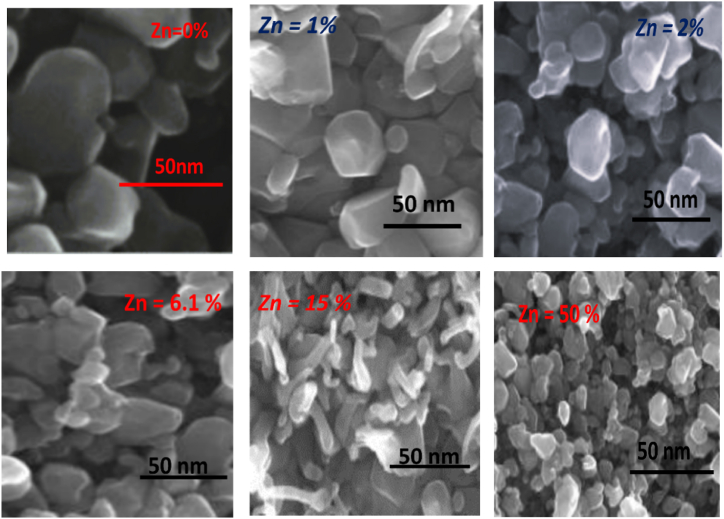
TEM image at 50 nm.

Fig. 2 shows that the transmission electron micrograph of p-type ${Zn}_{x}{Fe}_{1-x}S_{2}$ pyrite. From Fig. 2 we deduce that the structure of the obtained p-type ${Zn}_{x}{Fe}_{1-x}S_{2}$ pyrite shows granular and inhomogeneous structure. The grains have decreases size dimensions with increases of concentration of Zn, which is 68 nm (Zn=0%)64 nm (Zn=1%) 45 nm (Zn=2%), 37 nm (Zn=6.1%), 25 nm (Zn=15%), and 20 nm (Zn=50%). That confirm the good crystallinity.

**Fig. 3:**
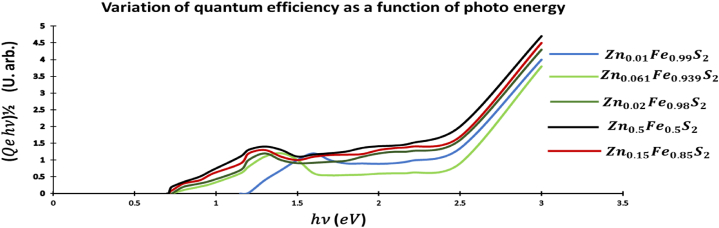
Plot hν versus ${\left(Qeh\nu \right)}^{1/2}$

Fig. 3 present the photon energy hν dependent Quantum efficiency as function ${\left(Qeh\nu \right)}^{\frac{1}{2}}$ of p-type ${Zn}_{x}{Fe}_{1-x}S_{2}$ pyrite for x(x=0,1,2,6.1,15and50%). We used mass spectrometry 350ICP- MS for this work. We observe three interesting phenomena: First the shift of the photocurrent toward bigger energies, in the energy range 1.8−2.2eV the Quantum efficiency increases, and the remarkable of a step-rising branch of ${\left(Qeh\nu \right)}^{\frac{1}{2}}$ graph curve above 2.2eV. This increase coincides with the energy gap (Eg=2.5eV) of p-type $ZnS_{2}$ pyrite [28]. From Fig. 1, we assume that Zn atom s substitute for Fe atoms in Iron pyrite. This substitution results the higher quantum efficiencies and the enlarger of the energy gap. The remarkable increase of the efficiency curve at 2.5eV show that Zn places in the $FeS_{2}$ pyrite change lattice electronically behaves like $ZnS_{2}$ pyrite.

**Fig. 4:**
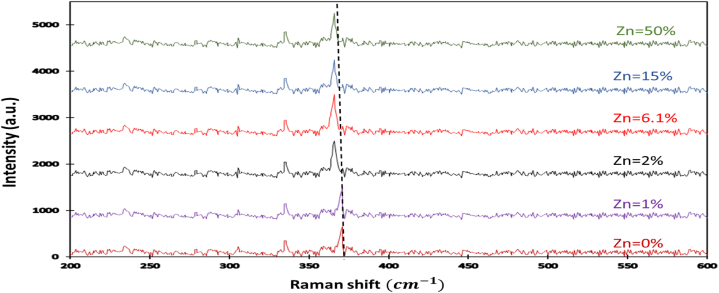
Raman spectra.

The Raman spectra of the prepared p-type ${Zn}_{x}{Fe}_{1-x}S_{2}$ are shown in Fig. 4. We have only two Raman peaks around 330 and 370 ${cm}^{-1}$ which defined $E^{LOW}$ and $E^{HIGH}$ corresponding to the in-phase stretching of the S–S dimer [48,49] and the vibrational mode of Fe–S [50]. No remarkable Raman peaks correspond to ZnS in the range of 200–600 ${cm}^{-1}\text{.}$ With increasing Zn percentage, the $E^{HIGH}$ a bleu shift of about 2.3 ${cm}^{-1}$ from 380 to 387 ${cm}^{-1}\text{,}$ while no clear change for $E^{LOW}$. All these indicate the increase of the lattice parameter and the S–S bond length with Zinc incorporation.

**Figure 5:**
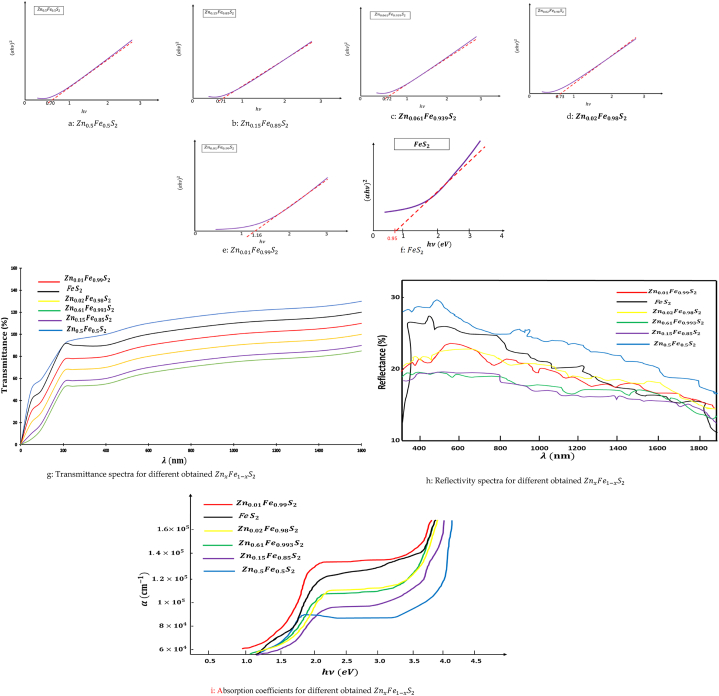
(b)${Zn}_{0.5}{Fe}_{0.5}S_{2}$.(b)${Zn}_{0.15}{Fe}_{0.85}S_{2}$.(c)${Zn}_{0.061}{Fe}_{0.939}S_{2}$.(d)${Zn}_{0.02}{Fe}_{0.98}S_{2}$.(e)${Zn}_{0.01}{Fe}_{0.99}S_{2}$.(f)$FeS_{2}$.(g)Transmittance spectra for different obtained $Zn_{x}Fe_{1-x}S_{2}$.(h)Reflectivity spectra for different obtained $Zn_{x}Fe_{1-x}S_{2}$.(i)Absorption coefficients for different obtained $Zn_{x}Fe_{1-x}S_{2}$.

Studying the optical band gap to study the impact of Zinc alloy on the type of state (direct or indirect) to the band gap of obtained ${Zn}_{x}{Fe}_{1-x}S_{2}$ pyrite. It is important to answer the question: after alloy of Zinc, will the band gap be direct or indirect? Because, if the ${Zn}_{x}{Fe}_{1-x}S_{2}$ pyrite will still a direct gap, which will be important for their use in photovoltaic cells as multispectral. Plots of ${\left(\alpha h\nu \right)}^{2}$ versus the photo energy hν are given in Figs. 5a–5f. These plots show straight line indicating that all ${Zn}_{x}{Fe}_{1-x}S_{2}$ and regardless of the percentages of Zn, has a direct band gap.

Figs. 5g and 5h show the plot of reflectance and transmission coefficients of our obtained samples taken in a spectral range between 240 nm and 1800 nm. A significant increase in transmittance was showed (Fig. 5g), but a decrease in the reflectance (Fig. 5h). The absorption coefficients are always high (Fig. 5i). Then, adding zinc in different percentages leaves a high optical absorption coefficient (α ≥ $10^{4}\;cm^{-1}$), which is the highest corresponding to ${Zn}_{0.01}{Fe}_{0.99}S_{2}$ which $\alpha >{1.4\times 10}^{5}$

**Fig. 6:**
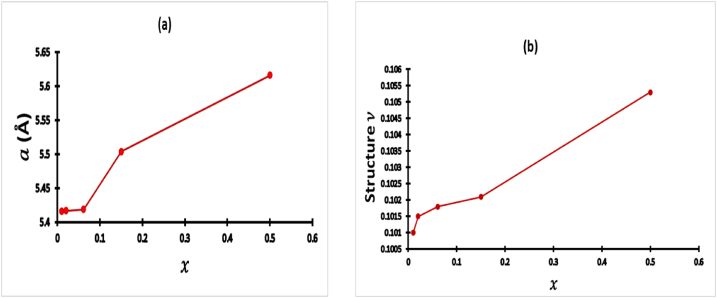
(a) Lattice constant a as a function x of Zn; (b) Structure υ as a function ofx of Zn;

Our experimental results show that the band gap increases for smallest concentration x=1% to 1.16eV and decreases for other x=2%,6.1%,15%and50% to 0.73,0.72,0.71and0.70eV. Hence, concentration x increases the band gap decreases implying it is more difficult to alloy pyrite with higher Zn concentration. Contrast to Pin Xiao et al. results [29], the band gap decreases for x greater than x=1% before x=6.2% because ${Zn}_{0.02}{Fe}_{0.98}S_{2}$ and ${Zn}_{0.061}{Fe}_{0.939}S_{2}$ have band gap values 0.73and0.72eV. An interesting finding of this experiment, the effect of Zn alloy concentration to lattice constant a, to position of the sulfur and the band gap. We have remarkable increases of lattice constant a, similarly to structure ν (parameter of position of Sulfur) increases with increases of Zn concentration, that is because Fe ions smaller than Zn ions. Our outcome is illustrated in Fig. 6a and b.

## 3 Electronic structure

The main reason that we are using LMTO-ASA method, is that it is an important tool in band structure calculations for compounds. We advise the reader to see Refs. [[30], [31], [32]], where they can find detailed description of such method. In our work, we use this technic because the calculations are done by DFT [33], utilizing the local density approximation [34] and numerical methods which based on the study of electron ion interaction in the pseudopotential approximation [35]. Moreover, the ASA Hamiltonian is mainly knowing by finding the potential parameters. Note that, the potential is between the logarithmic derivative $D_{v}$ at the sphere radius and the energy $E_{\nu }$ of the wave function φ. In general, it is enough to find one which can be used to find the other. Also, notice that knowing $D_{v}$ and $E_{\nu }$ makes finding the potential P a simple process using Eq. (4) [36,37].

(4) $$P\left(\varepsilon \right)=const\frac{D\left(\varepsilon \right)+l+1}{D\left(\varepsilon \right)-l}\approx {\left(\frac{\Delta_{l}}{\varepsilon -C_{l}}+\gamma_{l}\right)}^{-1}$$

where $C_{1}$, $\Delta_{1}$, and $\gamma_{1}$, are the “potential parameters” that parameterize P. $C_{1}$ corresponds to the band “center of gravity”, $\Delta_{1}$ is the “bandwidth” parameter and $\gamma_{1}$ is the “band distortion parameter”. In general, Small Parameterization is a good way to investigate band structure. We start with finding the potential parameter for all atomic spheres. The muffin-tin potential constant $V_{MTZ}$ was the crossing point of muffin-tin potential around Fe, Zn and S, ours $V_{MTZ}$ are presented in Table 2.

**Table 2:** The muffin-tin potential.

composition	$FeS_{2}$	${Zn}_{0.01}{Fe}_{0.99}S_{2}$	${Zn}_{0.02}{Fe}_{0.98}S_{2}$	${Zn}_{0.061}{Fe}_{0.939}S_{2}$	${Zn}_{0.15}{Fe}_{0.85}S_{2}$	${Zn}_{0.5}{Fe}_{0.5}S_{2}$
$V_{MTZ}$	−0.679774	−0.875619	−0.828333	−0.841527	−0.859493	−0.868554

The muffin-tin MT radii are 2.9387 for Zn, 2.9509 for Fe and 2.2017 for S. The initial sphere packing was equal to 79.2%, scaled to 87.9%. Note that, the empty spheres play an important role in reducing the number of iterations, as well as the reducing of the overlaps between the spheres centered at Fe, Zn and S.

The pyrite ${Zn}_{x}{Fe}_{1-x}S_{2}$ has a cubic crystal structure with space group $T_{h}^{6}\;\left(Pa\bar{3}\right)$. This structure has four metal atoms located at four positions and sulfur atoms in eight positions. The positions are presented in Table 3.

**Table 3:** Atomic position.

atom	Metal	Sulfur
Position	(0,0,0), $\left(0,\frac{1}{2},\frac{1}{2}\right)\text{,}$ $\left(\frac{1}{2},0,\frac{1}{2}\right)\text{,}$ $\left(\frac{1}{2},\frac{1}{2},0\right)$	$\pm \left(\frac{1}{2}-\nu ,\frac{1}{2}-\nu ,\frac{1}{2}-\nu \right)\text{,}$ $\pm \left(\nu ,-\nu ,\frac{1}{2}-\nu \right)\text{,}$ $\pm \left(\frac{1}{2}-\nu ,\nu ,-\nu \right)\text{,}$ $\pm \left(-\nu ,\frac{1}{2}-\nu ,\nu \right)\text{,}$

**Fig. 7:**
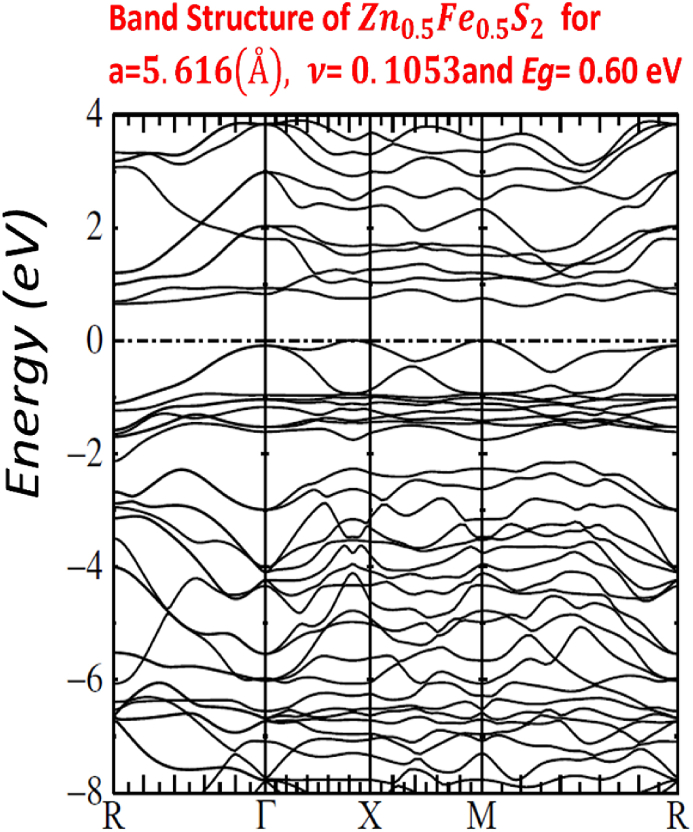
Band structure of ${Zn}_{0.5}{Fe}_{0.5}S_{2}$.

**Fig. 8:**
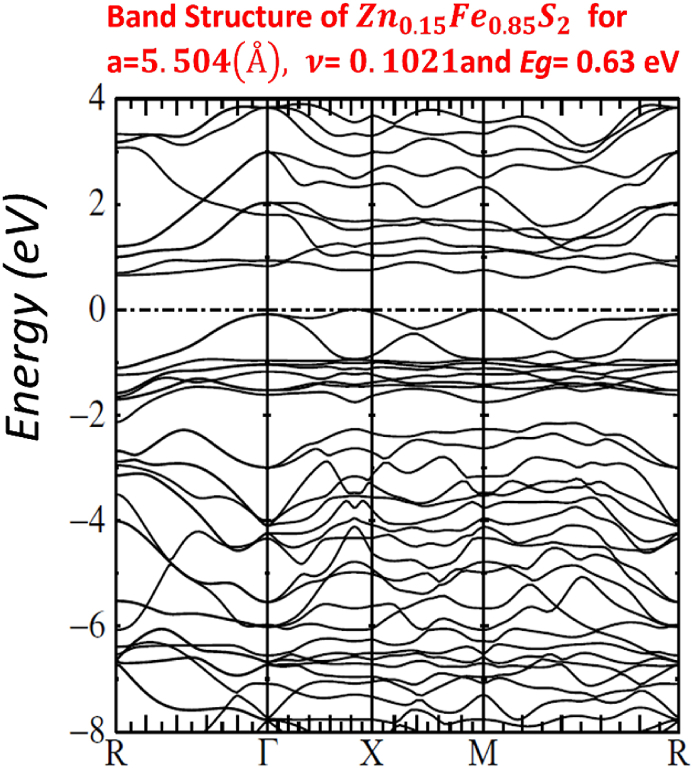
Band structure of ${Zn}_{0.15}{Fe}_{0.85}S_{2}$.

**Fig. 9:**
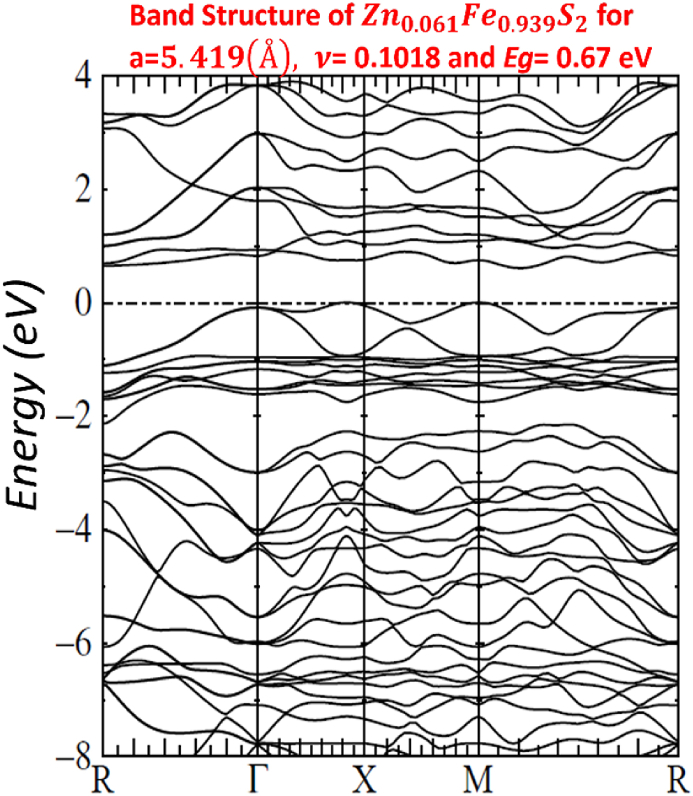
Band structure of ${Zn}_{0.061}{Fe}_{0.939}S_{2}$.

**Fig. 10:**
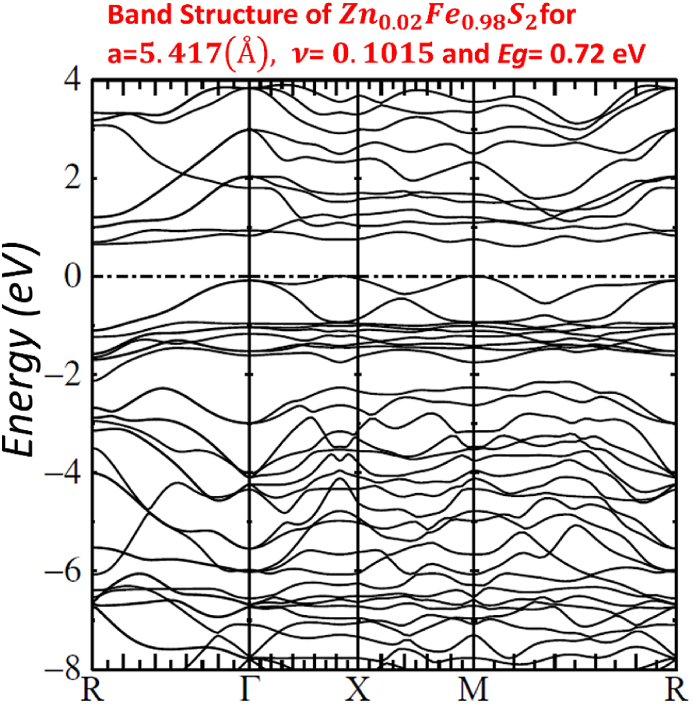
Band structure of ${Zn}_{0.02}{Fe}_{0.98}S_{2}$.

**Fig. 11:**
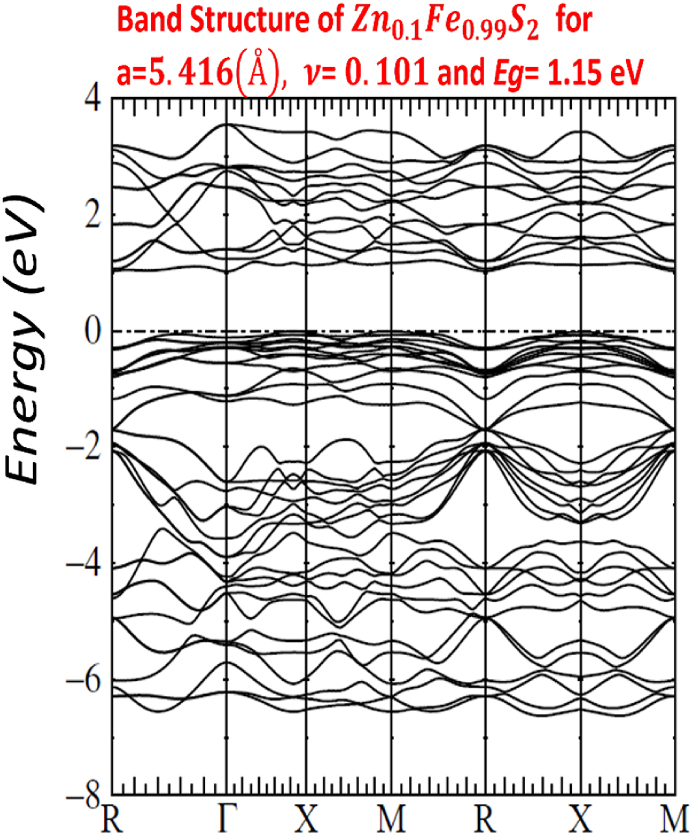
Band structure of ${Zn}_{0.01}{Fe}_{0.99}S_{2}$.

Our calculated LMTO-ASA energy bands are shown in Figs. 7–11. The five figures of ${Zn}_{x}{Fe}_{1-x}S_{2}$ present the band structure on a coarse energy. All figures show the area around the Fermi energy, which previous most details of the lowest conduction band and the highest valence band. All figures indicate that ${Zn}_{x}{Fe}_{1-x}S_{2}$ has a band gap semiconductor. The minimum conduction band and the maximum valence band are at Γ. Moreover, $FeS_{2},{Zn}_{0.01}{Fe}_{0.99}S_{2},{Zn}_{0.02}{Fe}_{0.98}S_{2},{Zn}_{0.061}{Fe}_{0.939}S_{2},{Zn}_{0.15}{Fe}_{0.85}S_{2}$ and ${Zn}_{0.5}{Fe}_{0.5}S_{2}$ have direct band gap found by LMTO-ASA. We were successful to enlarge the band gap, for ${Zn}_{0.01}{Fe}_{0.99}S_{2}\text{,}$ the value of the calculated gap is 1.15eV. This calculated gap is significantly agreeing with experimental gap 1.16eV, which proves the importance of using LMTO-ASA method to show the importance of experimental work. From our calculated gaps we can see that the band gap decreases to o.70 eV from x=2%. Also, we show that the band gap of ${Zn}_{0.01}{Fe}_{0.99}S_{2}$ is larger than the band gap obtained by Baodong Mao et al. [39] and these obtained by Pin Xiao et al. [29].

In Table 4, we list the values of direct band gap, empty bands $e_{c}$, filled bands $e_{v}$, minimum band conduction ${CB}_{\min}$, maximum valence band ${VB}_{\max}$, Fermi energy $E_{F}$ and gap experimental.

**Table 4:** band gap values.

composition	$FeS_{2}$	${Zn}_{0.01}{Fe}_{0.99}S_{2}$	${Zn}_{0.02}{Fe}_{0.98}S_{2}$	${Zn}_{0.061}{Fe}_{0.939}S_{2}$	${Zn}_{0.15}{Fe}_{0.85}S_{2}$	${Zn}_{0.5}{Fe}_{0.5}S_{2}$
Eg(eV)directexperimental	*0.95*	1.16	0.73	0.72	0.71	0.70
Eg(eV)direct LMTO−ASA	*0.92169*	1.15228	0.72264	0.67412	0.63605	0.60611
$E_{F}\left(Ryd\right)$	*−0.115652*	−0.0004635	0.210519	0.180514	0.158707	0.134831
$e_{c}={CB}_{\min}\;\left(Ryd\right)$	*−0.047880*	0.080091	0.263654	0.230082	0.205476	0.179398
$e_{v}={VB}_{\max}\;\left(Ryd\right)$	*−0.115652*	−0.0004635	0.210519	0.180514	0.158707	0.134831

**Fig. 12:**
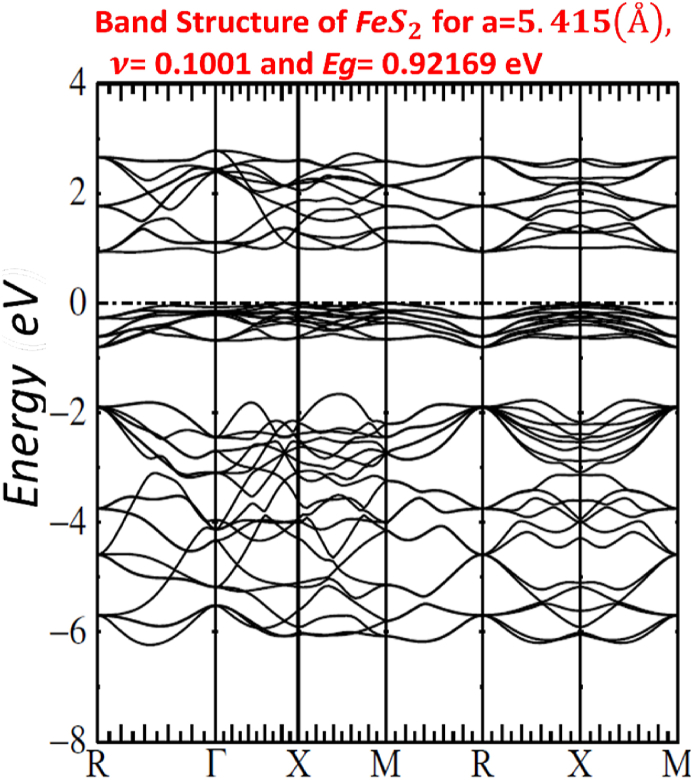
Band structure $FeS_{2}$.

All graphs show a full picture of both conduction bands and valence bands in an energy range −20eV and +8eV. Note that, the group of bands split between −18 eV and −12 eV are associated with bonding and antibonding pairs of orbitals on $S_{2}$. Fig. 12 of p*-type* $FeS_{2}$ pyrite shows the basic of S3p states with an additional with the Fe3d levels. The e_g_ orbitals are associated with the S3p states. Below the Fermi level, we have Fe3d t_2g_ bands, and above the Fermi level we have S3p and Fe 3d e_g_.

For other Figs. 7–11, They have S3s character and its states are predominant. From −9eV we have the state S3p with an addition to the Fe3d levels and Zn3d levels. This bands compose with bonding S3p and $Zn\;3d\;e_{g}$ and $Fe\;3d\;e_{g}$. The group of bands from −2eV to Fermi energy correspond to the S3p and $Zn\;3d\;t_{2g}$ and $Fe\;3d\;t_{2g}$. After, we have bands above the Fermi level, these bands have S3p and $Zn\;3d\;e_{g}$ and $Fe\;3d\;e_{g}$ character. In region of bands between −2eV to Fermi energy $E_{F}$, we noticed remarkable difference in distribution of bands between bands of ${Zn}_{0.01}{Fe}_{0.99}S_{2}$ and other for ${Zn}_{0.02}{Fe}_{0.98}S_{2},{Zn}_{0.061}{Fe}_{0.939}S_{2},{Zn}_{0.15}{Fe}_{0.85}S_{2}$ and ${Zn}_{0.5}{Fe}_{0.5}S_{2}$ that explained the surface behavior of Iron pyrite interface. As known Fe surface states are responsible to transfer of electronic charges to coordinating species such as iodide [38] and same mechanism can have predicted for Zn surface states that means our results for x=1% implying Zn surface states are involved in the electron transfer.

**Fig. 13:**
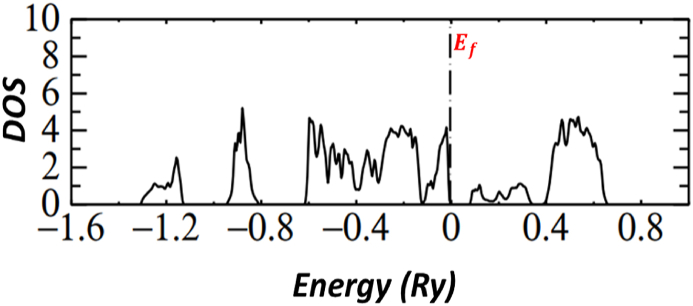
Density of states of ${Zn}_{0.01}{Fe}_{0.99}S_{2}$

Fig. 13 shows the density of states of ${Zn}_{0.01}{Fe}_{0.99}S_{2}$, calculated above and below the Fermi level. The density energy diagram started from −1.309182 Ry. The two peaks around −1.2 Ry and 0.8 Ry correspond to the dominant S contributions, the lowest of the valence bands. Also, between −0.6 Ry to 0 Ry is mostly dominated by S with a contribution from the dominant Zn and dominant Fe. The valence band maximum is between −0.1 and 0 below but very close to the Fermi level. And the conduction bands start from 0.080091 Ry above the Fermi level. That show the p-type pyrite of ${Zn}_{0.01}{Fe}_{0.99}S_{2}$. From these results we concluded that to enlarge the band gap of ${FeS}_{2}$ pyrite we must alloy with very small concentration of Zn. Note that Zn surface states involved in interfacial electron transfer where Zn surface states capture positive charge carries from the space charge layer during the electron transfer, which explain the significance of metal centered electron transfer which is a semiconductor photelectrochemistry.

Therefore, our work prove that we can enlarge band gap of ∼0.25eV for a very small amount f zinc ∼1% other than when the concentration increases for 2%,6.1%,15%and50% band gap decreases.

## 4 $Zn_{x}Fe_{1-x}S_{2}$ pyrite in photovoltaics

The synthesized p-type $Zn_{x}{Fe}_{1-x}S_{2}$ pyrite was evaluated for the application of photodetector devices; we used a device structure ITO/ $Cu_{2}O$/ $Zn_{x}{Fe}_{1-x}S_{2}$/Al. In this part we modeled the n-p heterojunction $Zn_{x}{Fe}_{1-x}S_{2}$ pyrite solar cell using a n-type window layer. Here a n-$Cu_{2}O$/ $p-Zn_{x}{Fe}_{1-x}$ heterojunction solar cell was created, and $Cu_{2}O$ was using as a n-type window layer. Since the density of states of $Cu_{2}O$ which has been largely studied for alloy and incorporation in material, studied its properties in device simulation [54].

**Figure 14:**
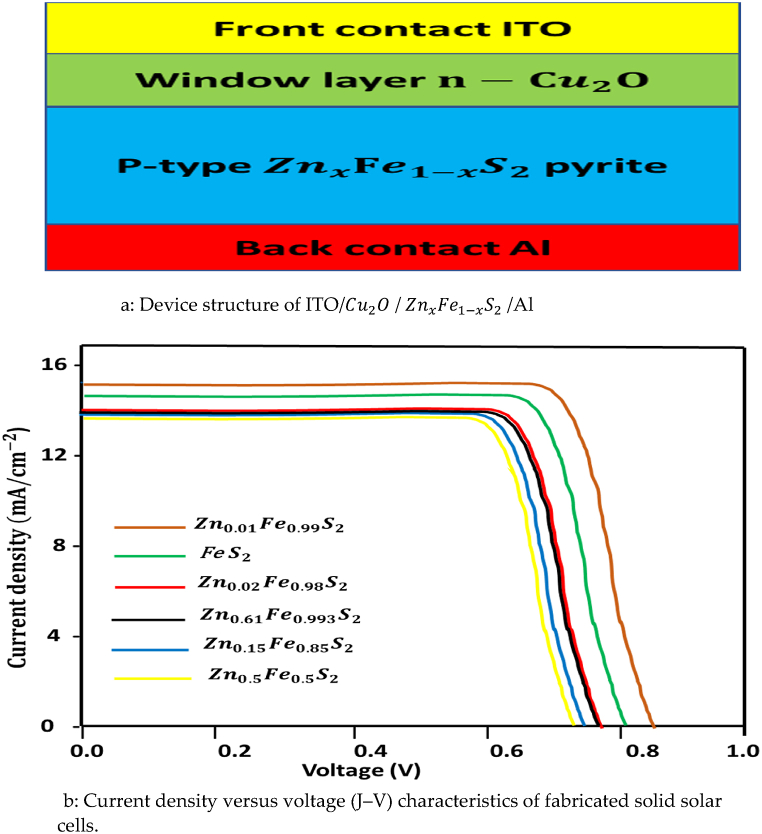
(a)Device structure of ITO/ $Cu_{2}O$/ $Zn_{x}{Fe}_{1-x}S_{2}$/Al.(b)Current density versus voltage (J–V) characteristics of fabricated solid solar cells.

The device structure is presented in Figs. 14a and 14b shows the band structure of the interface of n-$Cu_{2}O$/ $p-Zn_{x}{Fe}_{1-x}S_{2}$ at thermal equilibrium. We determined the current density condition by utilizing 0–1 V and found their photovoltaic characteristic.

Fig. 14b exhibit the current density voltage show the FF and J_sc_ for $p-type\;Zn_{x}{Fe}_{1-x}S_{2}$ solid solution which FF increase from 0.81 to 0.85 only for x = 0.01 and decreases from 0.81 to 0.71 for x for x increases from 0.02 to 0.5, and we observe J_sc_ increase from 15.10 to 15.50 also for x = 0.01 and decreases for other x = 0.02, 0.061, 0.15 and 0.5.

## 5 Conclusion

Our experimental and our calculations demonstrated that alloying Iron pyrite with small concentration 1% of zinc is an effective way to increase band gap of Iron pyrite, while conserve gap states also conserve the favorable electrical and optical properties. Our result show that p-type ${Zn}_{x}{Fe}_{1-x}S_{2}$ pyrite is a good candidate for photovoltaic application. Our experimental gap energy values agreed with our calculation s band gap. Through XRD and TEM, we examined our samples, which we show the grain size deceases with increases of percentage of Zinc. That confirm the good crystallinity

In summary, we have proved that the band gap of $FeS_{2}$ pyrite can be increased significantly if we add a tiny amount of zinc (Zn). The interesting part that our result was proved by using experimental technic and theoretical analysis as well. Also, we show that the band gap of ${Zn}_{0.01}{Fe}_{0.99}S_{2}$ is larger than the band gap obtained by Baodong Mao et al. and these obtained by Pin Xiao et al.

## Author contribution statement

Refka Sai, Ihab Shawish, Muaffaq M. Nofal, Eman A. Alghamdi: Conceived and designed the experiments; Performed the experiments; Analyzed and interpreted the data; Contributed reagents, materials, analysis tools or data, Wrote the paper.

## Funding statement

This research did not receive any specific grant from funding agencies in the public, commercial, or not-for-profit sectors.

## Data availability statement

Data will be made available on request.

## Institutional review board statement

Not applicable.

## Informed consent statement

Not applicable.

## Declaration of interest's statement

The authors declare no conflict of interest.
